# Molecular Prognosticators Guiding Fertility-Sparing Surgery in Early-Stage Endometrial Cancer: A Comprehensive Review

**DOI:** 10.3390/cancers17223602

**Published:** 2025-11-07

**Authors:** Saniyah Shaikh, Salsabil Haque, Hafsah Tajammul Khalifey, Halla Anas Samour, Ayesha Deed, Rutaba Mahereen, Noor Nabiha, Safwaan Shaikh, Lara M. Samhan, Mohammed Imran Khan, Ahmed Yaqinuddin

**Affiliations:** 1 College of Medicine, Alfaisal University, Riyadh 11533, Saudi Arabia; sanshaikh@alfaisal.edu (S.S.); sahaque@alfaisal.edu (S.H.); hkhalifey@alfaisal.edu (H.T.K.); hsamour@alfaisal.edu (H.A.S.); adeed@alfaisal.edu (A.D.); rmahereen@alfaisal.edu (R.M.); smshaikh@alfaisal.edu (S.S.); lsamhan@alfaisal.edu (L.M.S.); 2 Mamata Institute of Dental Sciences, Hyderabad 500090, India; noornabiharaf@gmail.com; 3 King Faisal Specialist Hospital and Research Center, Jeddah 21499, Saudi Arabia; mikhan@kfshrc.edu.sa

**Keywords:** endometrial cancer, fertility sparing surgery, molecular prognosticators, fertility preservation

## Abstract

**Simple Summary:**

Endometrial cancer is one of the most common cancers in women. The usual treatment, hysterectomy, results in complete infertility in women. For younger women who wish to preserve fertility, doctors sometimes use fertility-sparing surgery (FSS). However, the traditional criteria are limited to age, tumor features, and fertility goals. This review looks at how the integration of new molecular techniques can help improve patient selection and management. It aims to provide patients and doctors with a more personalized approach to treating early-stage endometrial cancer.

**Abstract:**

Background: Endometrial cancer (EC) is a common malignancy found among women. It is ranked as the 6th most common cancer among women and the 15th most common cancer globally. Increasing prevalence of several factors like obesity and other metabolic disorders have caused a growing trend of prevalence of endometrial cancer. The standard approach of treatment with excellent prognosis is total hysterectomy with bilateral salpingo-oophorectomy (TH/BSO). However, due to its drawback of complete infertility, newer approaches of fertility-sparing approaches are emerging to combat this challenge. Clinicians must choose the most suitable candidates for fertility-sparing surgery (FSS) using the present existing conventional criteria with regard to the patient’s age, tumor characteristics, and fertility goals. The limitations using the conventional criteria can be eliminated by refining the criteria with molecular prognostic factors to ease the candidate selection process for FSS. Methods: Relevant literature regarding molecular subtypes, hormone therapy sensitivity, clinical assessment, and guidelines pertaining to fertility preservation in EC were retrieved from several electronic databases and articles addressing the role of molecular profiling in predicting patient response, guiding patient selection, and/or informing the development of therapies for fertility preservation in early-stage EC, particularly in women of reproductive age were included. Primary focus was on areas of consensus, emerging trends, and evidence gaps that warrant further investigation. This review will assess the integration of molecular prognostic factors to refine the patient selection criteria and guide FSS in early-stage EC. We will present existing clinical criteria, ongoing clinical trials, limitations, and the advantages of integrating molecular data on patient selection, treatment safety, and fertility outcomes. Results: Four distinct molecular subtypes have been classified which includes POLE-mut, MMR-d, p53-abn and NSMP. POLE-mut subtype had excellent prognosis with >95% patients achieving complete remission with <2% recurrence rate followed by MMRd and NSMP with intermediate prognosis and lastly p53-abn with poor prognosis of 60–70% achieving complete remission and 30–40% having recurrence. The data highlights the clinical value of molecular classification in selecting appropriate candidates for fertility sparing surgery (FSS). Conclusions: There is a lack of integration of molecular subtypes for clinicians to choose candidates for FSS and this gap should be addressed. Further research must be performed to follow personalized medicine to refine their treatment plan.

## 1. Introduction

Endometrial cancer (EC) is a growing concern in women’s health worldwide. It is ranked as the 15th most common cancer globally and the 6th most common cancer among women. In 2022, there were 97,723 deaths reported due to EC [1]. In the United States, EC is now the 4th most frequently diagnosed cancer among women [2]. Traditionally, EC was seen as a disease affecting postmenopausal women. However, latest trends highlight a rising incidence in younger, premenopausal women aged under 50 [3,4].

These trends can be attributed to several factors. The rising prevalence of obesity and metabolic disorders like diabetes are amongst the strongest contributors, as both these conditions potentially influence hormone levels that promote abnormal growth of the endometrium [5,6]. Other potential contributing factors include low parity, early menarche, and lack of oral contraceptive use. While smoking is known to have multiple detrimental health effects, oddly it appears to lower the risk of EC in older women, though this certainly is not a recommended strategy due to its several known established harms [6]. Furthermore, with the increasing diagnosis in women of reproductive age, the significance of fertility preservation has grown remarkably. Numerous patients view the ability to have children post-treatment as their top priority, influencing their overall well-being and quality of life [7].

Early signs such as abnormal uterine bleeding help with timely diagnosis of EC before it progresses into later stages [8]. Among the younger women, a significant number of the cases are diagnosed early and at a less invasive stage. As a result, this allows for fertility preserving measures such as the use of less aggressive therapy like hormonal therapy without compromising safety and oncological outcomes [9].

Despite the benefits offered by alternative treatments, total hysterectomy with bilateral salpingo-oophorectomy (TH/BSO) remains the standard approach. Although it provides excellent cancer control, it results in complete infertility. In response, active surveillance or conservative management, such as high-dose progestins with or without hysteroscopic resection are now considered as viable approaches for carefully selected patients with early-stage, low-grade disease who wish to retain their reproductive capabilities [10]. These forms of surgical management are referred to as fertility-sparing surgery (FSS). That said, the selection criteria currently remain limited to histology and imaging findings which may not reflect the tumor biology [11]. This has sparked interest in molecular classification systems such as TCGA (The Cancer Genome Atlas) and ProMisE (Proactive Molecular Risk Classifier for endometrial cancer). These identify distinct subtypes of EC with different prognoses and treatment responses [12]. Therefore, utilizing molecular markers can improve risk identification and potentially enhance the safety of fertility-sparing approaches.

This review will assess the integration of molecular prognostic factors to refine the patient selection criteria and guide FSS in early-stage EC. We will present existing clinical criteria, their limitations, and how integrating molecular data can enhance patient selection, treatment safety, and fertility outcomes.

## 2. Methods

In this comprehensive narrative review, we summarize relevant literature, integrating both clinical and the translational aspects on molecular subtypes, hormone therapy sensitivity, clinical assessment, and guidelines pertaining to fertility preservation in EC. We employed a structured search to ensure broad and representative coverage of the literature.

We searched electronic databases such as the Cochrane Library, PubMed, Web of Science, and Google Scholar. A combination of medical subject heading (MeSH) and free-text keywords along with the use of Boolean operators (AND, OR) formed the search strategy. The keywords were, “endometrial cancer”, “fertility-sparing surgery”, “molecular markers”, “TCGA”, “ProMisE”, “POLE-mutation”, “mismatch repair deficiency”, “MMRd”, “p53 abnormal”, “hormonal therapy”, Lynch syndrome, “molecular classification”, and “progestin therapy”. To yield a broad but targeted set of relevant publications, searches primarily consisted of using these terms together with “endometrial cancer”. Keywords were used individually and in combination.

We considered articles that addressed the role of molecular profiling in predicting patient response, guiding patient selection, and/or informing the development of therapies for fertility preservation in early-stage EC, particularly in women of reproductive age. The acceptable types of studies were clinical trials, retrospective cohorts, review articles, expert opinion articles, experimental and observational research, and clinical guidelines. Only publications written in the English language were included. Surveys that were only restricted to advanced-stage disease, non-endometrioid histologies without addressing molecular profiling, and fertility preservation without addressing hormonal or molecular factors were outside the scope of the research.

The results were collated thematically in a narrative manner and located in the context of the wider therapeutic implication of fertility preservation in EC. The focus was on areas of consensus, emerging trends, and evidence gaps that warrant further investigation.

## 3. FSS in Early-Stage EC

FSS is generally reserved for carefully selected women with early-stage, low-risk endometrial cancer. The commonly accepted upper limits for eligibility include Grade 1 endometrioid adenocarcinoma that is confined to the endometrium (FIGO Stage IA, no myometrial invasion), with no evidence of extra-uterine spread (no adnexal involvement, no lymph node metastasis) on imaging or staging [10,13]. Candidates should undergo a thorough pre-treatment evaluation, such as MRI, to verify that the disease is limited to the uterus.

### 3.1. Conventional Criteria for Patient Selection

#### 3.1.1. Histological Criteria

Histological evaluation plays a key role when identifying candidates for FSS. Grade 1 endometrioid carcinoma, being the most well-differentiated and the most common form of EC, has been regarded as the best candidate for conservative treatment. More advanced tumors (G2 and G3) as well as non-endometrioid types, which include serous or clear cell carcinoma, are associated with poorer outcomes and a higher risk of recurrence, which precludes them from fertility preserving treatment modalities [14]. Furthermore, Grade 1 endometrioid tumors have a much lower risk of lymphovascular space invasion (LVSI) and extrauterine spread than higher-grade endometrioid tumors, reinforcing their candidacy for conservative management [15]. Tissue diagnosis is typically achieved using dilation and curettage (D&C) or hysteroscopic biopsy. However, a recent meta-analysis by Visser et al. evaluated 45 studies with 12,459 patients comparing preoperative endometrial sampling with hysterectomy specimens. The pooled agreement for tumor grade was 67%, with downgrading observed in 25% and upgrading in 21% of cases. These findings confirm that histologic discordance between biopsy and hysterectomy specimens can reach approximately 20–30% [16].

Therefore, preoperative biopsy is not effective in correlation with tumor grade, and its diagnostic performance is generally inferior to D&C.

#### 3.1.2. Imaging Criteria

Imaging is one of the best modalities for staging cancer. It is used to determine the absence of myometrial invasion and extrauterine spread which can preclude FSS. Magnetic resonance imaging (MRI) has emerged as the gold standard for detection of myometrial invasion, with sensitivity ranging from 80 to 90% and specificity up to 95% for identification of myometrial invasion [17,18]. Transvaginal ultrasound (TVUS) is also used to evaluate myometrial invasion, but its diagnostic accuracy is relatively lower, with a sensitivity of approximately 82% [19]. Both imaging modalities are crucial for the identification of deeper myometrial invasion and cervical involvement which are associated with higher recurrence risks. Although MRI has high predictive accuracy for assessing tumor depth of invasion, it cannot reliably detect microscopic LVSI, which contributes to recurrence [17]. According to recent studies, 24% of early-stage, low-grade (grade 1–2) endometrioid tumors showed LVSI, emphasizing the limitations of imaging in the recognition of a significant prognostic determinant [20]. Therefore, while imaging modalities can provide valuable information, they cannot fully encompass the tumor biology.

#### 3.1.3. Clinical Considerations

The selection of candidates to receive FSS entails tumor characteristics, patient age, and fertility goals. Young women aged 40 to 45 years with early-stage, low-grade tumors who wish to preserve their fertility are typically seen as suitable for conservative treatment. A thorough counseling approach covering the risks, RRs (recurrence rates), and requirement to maintain close follow-up is vital [21]. Hysteroscopic resection, alongside hormonal therapy, is increasingly recommended to remove localized tumors while keeping the uterus intact. Studies show that hysteroscopic resection followed by a norgestrel-releasing intrauterine device (LNG-IUD) or oral progestins can lead to oncologic outcomes similar to total hysterectomy, with pregnancy rates between 35% and 60% [13]. However, surgical resection can potentially cause intrauterine adhesions, which may affect fertility in the long-term [22].

During FSS for early-stage endometrial carcinoma, patients are monitored with hysteroscopic endometrial biopsy every 3–6 months during therapy, and every 6 months after the remission until pregnancy or definitive surgery, with MRI performed only if the recurrence is suspected. Close follow-up continues for at least 12 months post-remission. In the event of recurrence, repeat progestin therapy may be considered if disease remains uterine-confined, whereas definitive hysterectomy is indicated for progression, persistence after 6–12 months, or recurrence post-childbearing [10].

Despite these encouraging results high-quality evidence is limited, as noted in one of the meta-analyses that evaluated the effectiveness of the intra-uterine and oral progestins [23]. While most patients benefit from progestin therapy, response is not fully predictable [24,25]. The overall RR is 70%, with about 30% of patients experiencing resistance to the therapy [26]. Hence, this creates low adherence of the treatment in the non-responders [26].

**Figure 1:**
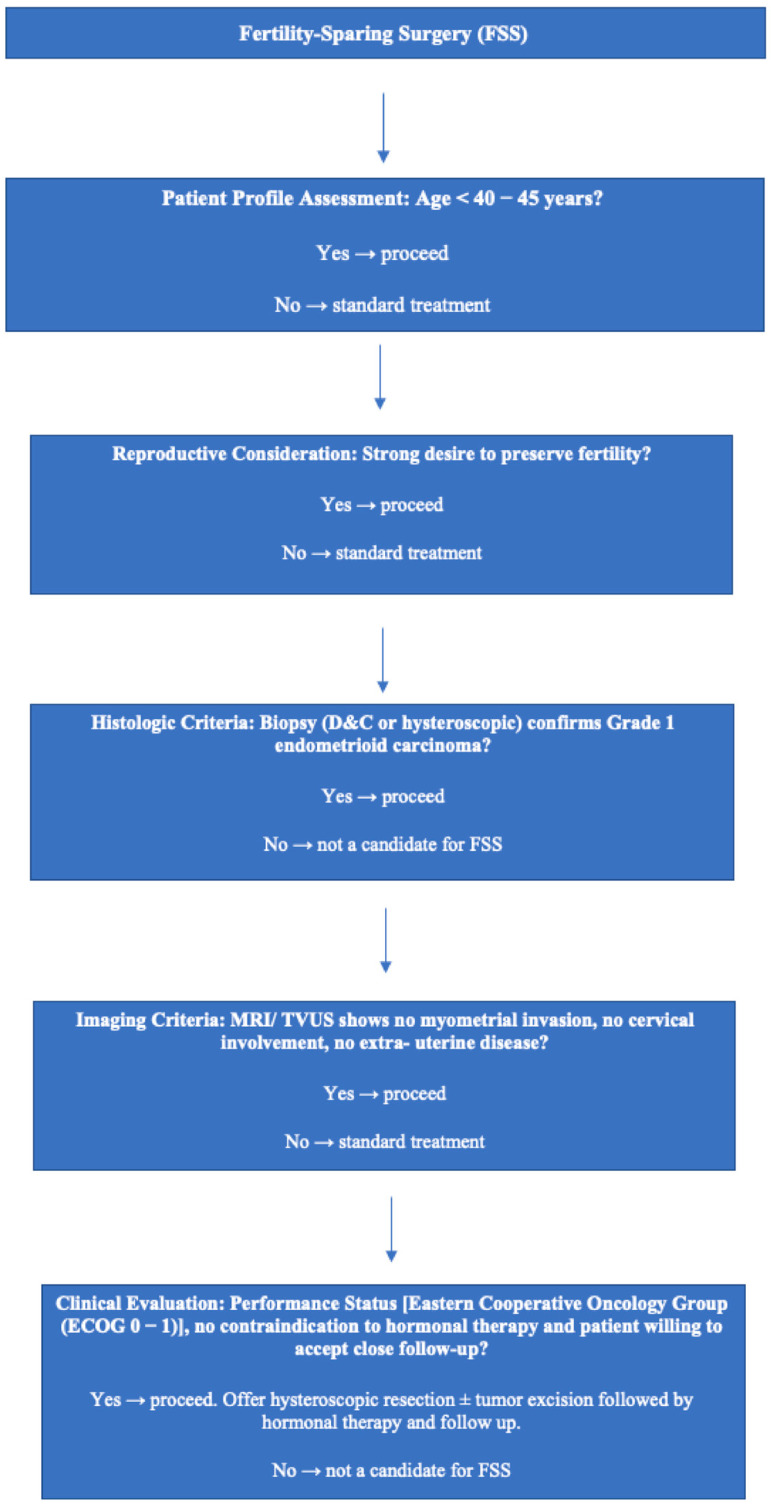
Flow diagram of patient selection for fertility-sparing surgery.

In conclusion, while the traditional criteria for FSS focus on histology, imaging, clinical factors, and provide a useful framework, they hold notable shortcomings. Figure 1 shows a flow diagram of patient selection for FSS. However, these criteria, based mainly on morphology and anatomical staging, do not fully capture tumor biology and may overlook important risk factors such as microscopic LVSI. As the field progresses, integrating molecular markers into the selection process could refine risk assessment, enhancing both oncological outcomes and fertility preservation.

### 3.2. Outcomes of FSS

The safety of FSS is pivotal, requiring a balance between disease control and fertility preservation. Although numerous studies have reported promising outcomes in well-selected patients, the risk of recurrence remains a significant concern. Table 1 compares the outcomes of various fertility-sparing treatment approaches.

#### 3.2.1. CR

The reported CR following hormonal treatment in carefully selected patients with early-stage, grade-1 EC ranges from 85 to 94% [31]. Approximately 93% of these individuals experience full recovery, typically within 7.5 months on average. Some individuals may experience full remission in as little as 4.5 months, while others may need up to 10.5 months or longer. This is consistent with current clinical practice, which uses progestin medication in conjunction with hysteroscopic surgery mainly for well-differentiated, early-stage illness in young women who want to preserve their fertility [32].

#### 3.2.2. RR

Recurrence remains a major challenge even after initial remission. RR following FSS are around 34.5%, with most occurring within the first two years of follow-up. Thankfully, most recurrences allow for repeat conservative treatment suggesting uterine-confined nature disease, although definitive surgery is often recommended upon recurrence [33].

#### 3.2.3. PR and LBR

Reproductive outcomes after FSS are generally encouraging but vary, largely depending on patient age and ovarian reserve. PR range from 33 to 65%, with LBR between 32 and 58% [31]. These rates are greatly reinforced by the use of ART which leads to higher PR after achieving CR [32].

#### 3.2.4. Long-Term Survival

Particularly, overall survival rates are high in this group, with 5-year survival exceeding 95% for women carefully selected for FSS based on favorable tumor characteristics [10]. Long-term data suggest that recurrence can generally be managed effectively but patients require close surveillance throughout their reproductive efforts [33].

## 4. EC Molecular Classification

### 4.1. The TCGA/ProMisE Classifiers

An important breakthrough in molecular characterization of EC happened in the year 2013, when TCGA introduced four molecular subtypes (Table 2): POLE-ultramutated, microsatellite instability (MSI), copy-number low (CN-L), and copy-number high (CN-H) [34]. They have used integrated genomic, transcriptomic, and proteomic characterization of 373 endometrial carcinomas using array- and sequencing-based technologies which have promising prospects for targeted therapy among patients [34,35]. However, its application in modern medicine was limited by the expensive and often inaccessible high-throughput sequencing procedure [36]. To facilitate clinical implementation, ProMisE has developed a modified classification, namely POLE-mutated (POLE-mut), mismatch repair deficient (MMR-d), p53 abnormality (p53-abn), and non-specific molecular profile (NSMP), using Sanger sequencing and immunohistochemistry (IHC) techniques.

However, there are several other barriers in the process of transitioning from research-grade classification to clinical reality. Validated immunohistochemistry and sequencing techniques are necessary for even surrogate classifiers like ProMisE, but these may not be standard in every facility [39]. Furthermore, skilled pathologists are needed to interpret molecular data, and inter-observer reporting variability may restrict cross-institution reproducibility [40]. These factors demonstrate that although molecular classifiers have a lot of potential, practical challenges need to be eliminated before they can be widely utilized to preserve fertility.

#### 4.1.1. POLE-Mut

The polymerase epsilon (POLE) gene plays a crucial role in the regulation of cell cycle checkpoints and chromatin modifications. Pathogenic somatic mutations in the POLE gene are responsible for initiating events of endometrial and colorectal cancers, seeing as they are sometimes found as early as in precancerous lesions [41]. Although a number of mutations in the POLE gene remain unknown, most of them occur in exons 9–14, specifically in exonuclease domains (EDM), collectively referred to as the “hotspot POLE mutations”, with P286R and V411L being the most prevalent [42]. In previous studies, the vast majority of POLE-ultramutated tumors presented as International Federation of Gynecology and Obstetrics (FIGO) stage I, grade 3 endometrioid carcinoma (EEC). Overall, around 8% of EC’s and 15% to 20% of FIGO grade 3 EEC’s show POLE EDM mutation and an ultra-mutated genotype.

Currently, de-escalation and even omission of adjuvant treatment is recommended for patients with EC with stage I–II tumors involving any of the EDM mutations, even in patients with other risk factors, classifying it as low-risk EC [43,44]. Thus, POLE EDMs may serve as a predictive biomarker of favorable prognosis. Possible proposed mechanisms for improved prognosis include high mutation burden, increased base substitutions [45] increased neoantigens, and elevated CD8+ tumor infiltrating lymphocytes [46].

#### 4.1.2. MMRd/Microsatellite Instability-High (MSI-High)

Another specific molecular pathway involved in EC is MMRd and MSI. MMR (Mismatch repair) proteins (MLH1, MSH2, MSH6, PMS2) are responsible for correcting DNA replication errors [47]. Loss of function, be it due to epigenetic silencing or germline mutations, causes increased mutational rates and genomic instability [48]. This results in MSI-H tumors in the colon, endometrium, and ovaries, more commonly referred to as Lynch syndrome [48]. MMRd/MSI-H tumors are most commonly endometrioid in their histology and have intermediate prognosis [49,50].

Although studies have found inconsistent correlations between MSI-H status and adverse histopathologic parameters, the presence of MMR deficiency has emerged as an important predictor of response to immunotherapy. In 2019, the European Society for Medical Oncology (ESMO) recommended d-MMR/MSI-H evaluation as a diagnostic tool in patients with Lynch Syndrome, utilizing IHC or molecular polymerase chain reaction-based (PCR-based) tests to detect d-MMR and MSI-H status, respectively [51]. D-MMR/MSI-H status was also approved to select EC patients that could benefit from immune checkpoint inhibitors such as dostarlimab [52,53].

#### 4.1.3. No Specific Molecular Profile (NSMP)

The NSMP subgroup encompasses tumors lacking POLE-mut, MMR deficiency, or p53-abn and are poorly understood due to their variable prognoses [54]. NSMP ECs are typically hormone receptor-positive and predominantly classified into low- to intermediate grade ECs. The prognostication in this group is heterogeneous and mainly depends on clinicopathologic outcomes such as FIGO grade, stage, LVSI, and estrogen receptor (ER) status [54].

Recent data suggest that ER-positive NSMP tumors are associated with reduced recurrence risk compared to their ER-negative counterparts [54]. Therefore, there is a possibility of further risk stratification in this molecularly ambiguous population using ER IHC to identify individuals that can be treated with fertility-sparing approaches. Moreover, the majority of NSMP tumors tend to express high ER (>80%) and progesterone receptor (PR), which makes them excellent candidates for a progestin-based regimen in fertility-preserving patients [55].

#### 4.1.4. p53-Abn

TP53 gene mutation is common in high-grade serous tumors of the ovaries, fallopian tubes, peritoneum, and endometrium [56]. These tumors are typically high-grade serous or high-grade EEC and are associated with an aggressive clinical course, poor prognosis, and reduced overall survival [57]. Data from the Post Operative Radiation Therapy in Endometrial Carcinoma (PORTEC) trials have demonstrated p53 mutations to be a poor prognostic marker, independent of the traditional risk factors [58]. Consequently, the presence of p53-abn is now considered an indication for escalation of adjuvant therapy, reinforcing the critical role of molecular classification in therapeutic decision-making.

**Figure 2:**
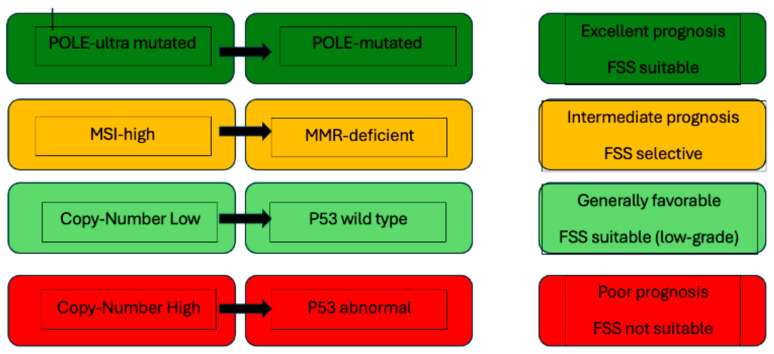
Schematic diagram: TCGA/ProMisE molecular subtypes with prognostic implications and FSS suitability.

Table 2 and Figure 2 summarize the prognosis and implications of FSS of the various molecular subtypes.

### 4.2. Molecular Markers and Hormonal Therapy Response

The presence of expression of ER and PR continues to be one of the pillars in terms of prognosis and treatment of EC. High-grade tumors frequently exhibit loss of one or both receptors [59], correlating with poorer prognosis and reduced responsiveness to hormonal therapy (progestins). Despite extensive investigation, consistent molecular predictors of complete response to progestins remain elusive. Meta-analyses suggest that PR, PRβ, and ERα are associated with higher regression rates, whereas glandular PRβ expression may portend a higher risk of recurrence [55,60].

Besides hormone receptors, several molecular alterations have emerged as potential biomarkers of prognosis and response to therapy. PTEN-deficient tumors exhibit enhanced proliferative signaling and may demonstrate resistance to certain therapies. Accordingly, inhibitors of the PI3K/AKT/mTOR pathway are being explored as targeted strategies [61]. Similarly, mutations in ARID1A, a chromatin remodeler, are prevalent in both endometrioid and clear cell carcinomas, with a higher frequency in high-grade subtypes. These alterations contribute to aberrant gene expression and have been associated with MMR deficiency and MSI [62]. Further research on the mechanism of action of ARID1A is required to devise new diagnostic, therapeutic, and prognostic strategies [61]. Other notable alterations include KRAS mutations, predominantly in the endometrioid subtype, activating the MAPK/ERK pathway, and CTNNB1 mutations, leading to nuclear accumulation of β-catenin and Wnt pathway dysregulation. These molecular events promote tumorigenesis through enhanced proliferation, survival, and invasiveness [61].

Despite these advances, no single biomarker has yet demonstrated consistent predictive values for hormonal therapy response. Ongoing efforts to delineate the molecular determinants of treatment response and resistance will be essential to achieving personalized, biology-driven therapy in EC.

### 4.3. Real-World Prevalence of Molecular Subtypes in FSS Candidates

Current real-world research demonstrates that the molecular subtypes identified by

TCGA and surrogate classifiers such as ProMisE can be identified in early-stage endometrioid-dominant EC, the primary population evaluated for FSS. The prevalence of these subtypes varies across cohorts (Table 3), highlighting the biological heterogeneity of seemingly low risk-tumors [58,63,64].

- TP53-wild-type (52%)/NSMP: (43–52%)
- TP53-mutant/p53-abn: (18–22%)
- POLE-mut: (5–10%)
- MSI-high/MMRd: (20–28%)

Although low-grade, early-stage endometrioid tumors are the primary criteria for traditional FSS eligibility, our study shows that a significant number of these patients have undesirable molecular characteristics. NSMP, a subtype with an intermediate and variable prognosis, accounted for 61.7% (589/954) of the 954 low-grade endometrioid cases. More importantly, there were also p53-abn and MMRd tumors, suggesting that histological appearance might not fully reflect the biological risk [66]. On the other hand, certain patients with high-grade tumors who would normally be excluded from FSS may fit into favorable molecular categories, especially POLE-mut ECs, and therefore become candidates for more conservative treatment.

Furthermore, concurrent TP53 mutations may be present in up to 42% of POLEmut ECs, which could complicate their IHC profiles and highlight the need for genomic confirmation in some circumstances [67].

However, the shift raises questions about cost and accessibility. Tumor-normal sequencing panels or WES-based classifiers are becoming increasingly practical in high-resource centers, but their availability is constrained by the infrastructure and cost in low-resource settings [40,68]. Additionally, pathology expertise determines the quality of testing, and differences in interpretation could cause patients to be misclassified or delayed. Therefore, even though there is evidence to support molecular testing in theory, ensuring equitable access is still a significant challenge [69].

Moreover, recent updates, such as the 2023 FIGO staging, now recommend integrating molecular subtypes (POLE-mut, MMRd, p53abn, NSMP) with traditional histopathological factors (grade, myometrial invasion, lymphovascular space invasion [LVSI]) to improve prognostic accuracy and guide individualized treatment, including fertility-sparing options. Molecular classification provides additional, sometimes superior, prognostic information, but does not fully replace the need for detailed histopathological assessment, especially for features like myometrial invasion and LVSI, which remain critical for risk stratification and treatment decisions [70].

## 5. Clinical Applications of Molecular Markers in Fertility-Sparing Decision-Making

The impact of molecular profiling on detecting molecular prognosticators and in the early management of endometrial cancer has emerged as a powerful tool, especially in guiding FSSs (Table 4). Multiple molecular classification systems have been developed, categorizing ECs into distinct subtypes based on their molecular features [9,36].

### 5.1. Cases

- Case 1: POLE-mutated, grade 2—Expanding FSS eligibility

POLE-mut grade 2 EC is one of the unique molecular subtypes and usually has a good prognosis [34]. In EC, there are five confirmed pathogenic somatic POLE-EDM mutations that are located at codons 286, 411, 297, 456, and 459. These are called “hotspot” mutations and have high mutational burden and increased immunogenicity [34,44]. These tumors may be considered for de-escalation of therapy and FSS, even in the presence of other risk factors [44,75,76,77]. Multiple studies report CR ranging from 70 to 74% and RR comparable to grad 1 EC. Patients typically receive progestin therapy ± levonorgestrel-releasing intrauterine devices (LNG-IUS) or hysteroscopic resection [78].

- Case 2: MSI-H, PR-negative—Relapse risk and low response risk

MSI-H tumors, often PR-negative, exhibit high mutation rate but variable immune responsiveness. They are associated with a higher risk of relapse and poorer responses to progestin therapy so alternative treatments (immune checkpoint inhibitors, CDK inhibitors) may be considered [79,80,81].

- Case 3: NSMP + PR-positive—Likely to benefit from hormonal therapy.

NSMP EC portrays intermediate prognosis and is frequently hormone responsive, particularly when PR expression is high (90–100%). High PR expression in NSMO EC reflects hormone responsive, less aggressive biology with better survival, lower recurrence, and favorable response to progestins [21,54,82].

- Case 4: p53-abn/CN-high—Poor prognosis

P53-abn tumors exhibit aggressive histology and are generally resistant to conservative therapy. FSS is typically contraindicated, and standard approach is recommended [11,71,73].

The relevance of increasing FSS eligibility through molecular stratification (Table 5) depends on the feasibility and reliability of testing. Barriers include high sequencing costs, lack of access to molecular pathology in non-tertiary hospitals, and requirement for highly qualified pathologists for precise interpretation [36,83,84]. Without addressing these, clinical use may be limited, increasing patient care inequities.

One significant gap is that the majority of guidelines are descriptive rather than operational, frequently mentioning the potential benefits of molecular classifiers without specifying how they should actually change fertility-sparing choices [85]. Clinical trial frameworks like the PORTEC series have shown how adjuvant therapy decisions can be improved by molecular stratification. For example, PORTEC-3, and PORTEC-4a specifically included POLE-mut and p53-abn status in risk stratification, demonstrating that molecular profiles are more accurate outcome predictors than conventional histopathologic standards [58,88]. Similarly, the ProMisE classifier has been prospectively validated in several cohorts, demonstrating its clinical efficacy and repeatability in differentiating between low-risk (p53-abn) and favorable (POLE-mut, NSMP) categories [11,40].

Among the molecular subtypes, the most robust evidence for safely expanding fertility-sparing surgery (FSS) eligibility is observed in POLE-mutated and NSMP tumors. POLE-mut ECs demonstrate exceptional prognosis and low recurrence even in grade 2 disease, supporting consideration for de-escalation and FSS despite higher histologic grade [36,44]. Similarly, NSMP, especially ER/PR-positive tumors, have low recurrence rates and excellent hormonal reactivity, confirming their usefulness for conservative treatment [43,68]. On the other hand, p53-abn tumors are still not recommended for FSS because of their aggressive biology, while MMRd ECs show intermediate results that call for careful monitoring [57].

### 5.2. Ongoing Clinical Trials

Fertility rates among women over 35 have increased by 67%, mostly because of enhanced ART and patient driven-decision-making. To preserve fertility, long-term progestin therapy in conjunction with rigorous clinical monitoring may be an appropriate therapeutic option for carefully chosen patients with grade 1 atypical endometrial hyperplasia. RR ranges from 10% to 47%, while CR ranges from 53% to 84%. The search for pathologic and clinical indicators that might accurately forecast progestin treatment response and future recurrence risk is still ongoing. Integrating molecular classification with clinical and pathological factors may further refine patient selection [89].

The mainstays of fertility-preserving therapy are LNG-IUS and oral progestins, which have been shown to be safe and effective for women with early-stage EC and little to no myometrial invasion. High-dose oral progestins are generally well-tolerated, though side effects such as blood clots, weight gain, and headaches may impact patient compliance. Current clinical trials are assessing the efficacy of LNG-IUDs, whether used alone or in conjunction with oral progestins or metformin, in younger women diagnosed with low-grade EC.

Bariatric surgery has been shown to greatly reduce the risk of EC in women with obesity. While promising, its broader effects on fertility and long-term outcomes require further investigation.

Further investigation is warranted to validate the utility of IHC markers and serum CA125 levels as predictive tools for treatment response. Preliminary studies incorporating molecular markers, such as p53 mutations and MMR deficiencies, have shown promise in refining risk stratification for fertility-sparing management. Ongoing research is also focused on identifying endometrial biomarkers capable of predicting therapeutic resistance, which could enhance patient selection and optimize clinical outcomes [90].

Multiple prospective studies and randomized controlled trials (RCT) are currently in progress to determine the most effective therapeutic strategy for EC patients seeking fertility preservation (Table 6).

The study (Clinical trial NCT02990728) under evaluation is the effectiveness of the LNG-IUS alone and in combination with metformin, a fertility-preserving therapy against grade 1 endometrioid endometrial carcinoma.

Clinical Trial NCT03241914 is a RCT assessing that megestrol acetate and LNG-IUS are not inferior in restoring normal endometrial histology in the early stages of EC in comparison to megestrol acetate alone.

Clinical Trial NCT03463252 would evaluate the effectiveness of LNG-IUS in the fertility-sparing treatment of atypical endometrial hyperplasia and EEC, by measuring pathological outcome and subsequent pregnancies [91].

Artificial intelligence (AI) constitutes a robust analytical instrument for interrogating complex datasets, facilitating the identification of subtle and multifaceted patterns that remain undetected through conventional human analysis. Expanding the integration of AI methodologies within clinical research and practice holds significant promise for improving diagnostic accuracy and clinical outcomes in the context of gynecologic malignancies. The integration of AI into histopathological workflows holds transformative potential by enabling the rapid and precise analysis and classification of intricate cellular architectures. The advancement may significantly enhance the efficiency and accuracy of pathological assessment. Across the domains of histopathology, medical imaging, and multi-omics, AI has exhibited substantial potential to improve diagnostic precision, minimize interpretive variability, and accelerate the clinical decision-making process [92,93].

Current evidence indicates that MMRd ECs are associated with limited responsiveness to progestin therapy and an elevated risk of recurrence, underscoring the need for alternative therapeutic strategies, including the investigation of targeted treatments, which remain an underexplored area. POLE-ultramutated tumors are linked to favorable outcomes; however, the oncologic safety of hysteroscopic resection as a standalone fertility-preserving approach requires further clinical validation. In contrast, tumors harboring p53-abn demonstrate poor prognostic profiles, casting doubt on their appropriateness for conservative management. Tumors lacking a defining molecular alteration (p53 wild-type/NSMP) exhibit moderate prognoses but encompass significant biological heterogeneity, necessitating the identification of more discriminative biomarkers to stratify risk and tailor therapy. Addressing these gaps is critical for refining molecular criteria and informing evidence-based selection for fertility-sparing treatment in EC [94].

Molecular stratification is gradually being incorporated into fertility-sparing treatment through ongoing RCTs. Early research, like the feMMe trial (NCT01686126) and KGOG trial (NCT01594879), primarily examined clinical efficacy; however, more recent cohorts, like NCT03538704, use TCGA/ProMisE classifiers to forecast treatment response and recurrence risk. Furthermore, a number of studies now link oncological safety with reproductive outcomes by including PR and LBR as secondary endpoints [13,40,74,87].

## 6. Challenges, Gaps, and Future Directions

### 6.1. Current Challenges

#### 6.1.1. Limitations of Current Evidence

Molecular prognosticators remain limited in clinical utility due to several persistent challenges, including a lack of robustness and reproducibility across datasets, limited biological interpretability of gene signatures, insufficient validation in independent cohorts, and difficulties in integrating complex molecular data with clinical variables [95]. Many established prognostic gene expression signatures do not provide clear biological explanations or causal insights into disease mechanisms, and there is often little overlap between gene lists from different studies, with some random gene sets performing comparably to published signatures [96]. Most of the current evidence base for fertility-sparing treatment in EC is limited to retrospective cohorts and further prospective validation is needed to determine the best treatment for patients [27]. Additionally, only a few molecular signatures have reached clinical implementation, hindered by high costs, technical complexity, and lack of standardization. These issues are compounded by disparities in access to molecular testing and underrepresentation of diverse populations in foundational studies, which limit the generalizability and equity of these tools [97]. In addition, current guidelines and practices are mostly linked to type 1 EC, which is a minimally invasive endometrioid cancer, while there is no supporting evidence on type II EC which is deeply invasive, has worse prognosis, and is of non-endometrioid histology, suggesting oncologic safety still remains a concern in non-endometrioid and high-grade cancer [98]. Despite having the best evidence and guidelines, fertility-sparing treatments are recommended for grade 1 EC [10]. This indicated oncologic safety cannot be guaranteed in high- grade, non-endometrioid types.

Recent evidence shows that molecular subtyping (ProMise/TCGA) has a prognostic value in fertility-sparing treatment in early-stage endometrial cancer. MMRd/MSI-H tumors exhibit an intermediate response with a higher recurrence in combined series, p53-abn tumors show poor oncologic outcomes, whereas NSMP tumors show highest response to progestin based FST. Moreover, the current evidence is mainly based on retrospective cohorts and systematic reviews and lacks prospective studies and randomized trials. As a result, even though molecular classification improves FST risk stratification, most of the guidelines are still based on poor-quality and heterogenous available data [36,68,99].

#### 6.1.2. Limitations of Clinical Application

Beyond biological uncertainty, practical obstacles hinder widespread clinical adoption of molecular prognosticators in fertility-sparing treatment. Firstly, cost-effectiveness remains a major obstacle [69]. Multi-omic profiling and sophisticated sequencing platforms are costly and often unavailable in resource-limited settings. Variability in reimbursement policies further limits patient access [87,100]. Secondly, infrastructure limitations exist. While testing for POLE, MMR, and p53 may be routine at large academic centers; however, community and rural hospitals often lack the necessary equipment, delaying or preventing molecular stratification [40,83]. Lastly, pathology expertise is critical to successful implementation. Variability in IHC reporting and interpretation can reduce consistency across institutions, and subspecialized gynecologic pathologists are essential [69,101].

Despite encouraging evidence, molecular integration into fertility-preserving techniques is not yet generally possible due to these practical considerations. Therefore, it is crucial for equitable adoption to address these real-world issues through standardized procedures, capacity-building, and cost-cutting measures.

### 6.2. Moral and Psyche Issues on Molecular Stratification of FSS

A loss of functional tumor suppressor gene PTEN is associated with increased EC risk, while Lynch syndrome also increases risk of EC and ovarian cancer, influencing a patient’s choice towards conservative, fertility-preserving options.

Women generally value fertility and parenthood but experience fear and anxiety after a cancer diagnoses. Potential complications associated with fertility preservation may further impact acceptance of intervention [102].

The ProMisE classifier provides a clinically practical stratification scheme; however, its role in fertility-preserving treatment approaches is still not well established, and guidelines offer limited actionable clinical recommendations for use in this area [103].

Fertility-sparing options must be integrated into shared decision-making, particularly considering the psychological impact of fertility loss following standard oncologic procedures [104]. Onco-fertility care should balance preservation of maternal life, fetal well-being, and early minimally invasive evaluation of reproduction and overall health. Developing a multidisciplinary, holistic approach to address such complex and conflicting priorities is a major clinical and ethical challenge [13].

### 6.3. Future Directions

Future research in fertility-sparing management of early-stage endometrial cancer should prioritize biomarker-driven, stratified RCTs that integrate molecular subtyping (POLE, MMRd, p53-abn, NSMP) with validated predictors such as PR expression and early Ki-67 suppression [81,88].

Incorporating AI tools like im4MEC and deep learning-based histopathology, alongside ctDNA assays, offers the potential for real-time, noninvasive monitoring of treatment response and recurrence risk [91].

Factorial designs combining progestin-based therapy with metabolic interventions (e.g., weight loss, metformin) are especially relevant for obese or insulin-resistant patients. Crucially, future trials must embed prospective biomarker validation and ensure reproducibility across laboratories [17,105].

**Figure 3:**
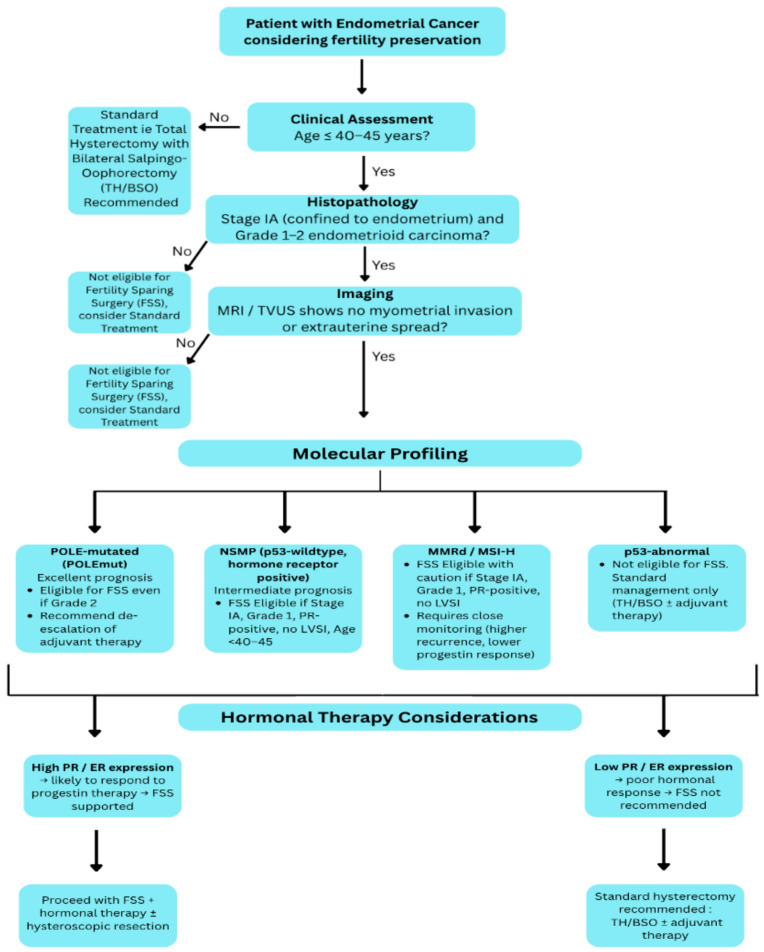
Proposed decision-making algorithm integrating molecular classification with traditional histopathology for FSS eligibility.

While these technologies promise earlier detection, refined risk stratification, and personalized treatment planning; cost, accessibility, and standardization remain major challenges for broad clinical implementation. Figure 3 demonstrates a proposed decision-making algorithm integrating molecular classification with traditional histopathology for FSS eligibility. Advancing toward biomarker-driven, stratified RCT that integrate molecular subtyping with validated predictors of response is essential to deliver truly personalized fertility-preserving management in early-stage EC.

## 7. Conclusions

As EC increasingly affects younger individuals with reproductive potential, the evolution of its classification from purely histopathological criteria to a molecular informed framework is not merely an academic pursuit, but a clinical imperative. While retrospective data supports the role of molecular subtypes such as POLE-mut, NSMP, and MMRd in refining fertility-sparing management, these studies are inherently limited by design. This evidence gap leaves clinicians navigating a nuanced gray zone for patients whose fertility is at stake.

To advance the field, prospective, subtype-specific RCTs are required to establish oncologic safety and reproductive efficacy. Adopting shared decision-making models, integrating risk stratification tools, and involving transdisciplinary tumor boards. Equally important, equitable access to molecular testing and fertility preserving care must be addressed, particularly in settings where cost, infrastructure, and expertise remain barriers.

Ultimately, the future of fertility preservation in EC relies on three intersecting trajectories: scientific precision, clinical pragmatism, and psychosocial empathy. Advancing research in this direction will bridge current knowledge gaps, empower precision medicine, and transform a clinical challenge into a platform for innovation and equity. The next decade will decide whether fertility-sparing therapy in EC becomes a model of precision oncology or remains constrained by inequity and evidence gaps.

Fertility-preserving programs in EC need to include quantifiable results corresponding to the suggested pillars to operationalize these future directions. Molecular testing uptake rates, subtype-specific recurrence-free survival, and the relationship between molecular markers and therapy response are all ways to assess scientific precision. Metrics like cost-effectiveness, time-to-treatment initiation, availability of molecular diagnostics, and compliance with evidence-based FSS guidelines can all be used to evaluate clinical pragmatism. The assessment of psychosocial empathy should be performed with established patient-reported outcome measures (PROMs), such as mental health indices both during and after treatment, fertility-related quality of life, and decisional satisfaction. Embedding these indicators within future clinical trials and registries would enable a holistic assessment of both oncologic and patient-centered success.

## Figures and Tables

**Table 1:** Comparison of oncologic and reproductive outcomes of fertility-sparing treatment modalities in early-stage EC.

Treatment Modality	Complete Response Rate (CR)	Time to CR (Months)	Recurrence Rate (RR)	Pregnancy Rate (PR)	Live Birth Rate (LBR)	Key Notes	References
Oral progestins	76%	-	28%	-	17%		[27]
LNG-based therapies	63.4%	More than 24	29.6%	37.9%	39.3%	It is a viable conservation modality	[28]
Hysteroscopic resection + progestin	90%	3–6	17%	49%	45%	This treatment modality showed overall improved response rates of pregnancy and live births.	[29]
Combination with ART	-	4.2 ± 2.5	16.7%	78.7%	53.2%	Beneficial for pregnancies and did not have a negative impact on cancer recurrence.	[30]

**Table 2:** TCGA/ProMisE molecular subtypes with prognostic and fertility-sparing implications.

Molecular Subtype	ProMisE Equivalent	Prognosis	Common Features	Implications for Fertility-Sparing Surgery (FSS)	Reference
POLE-ultramutated	POLE-mut	Excellent	High-grade tumors, younger patients, low recurrence risk	Despite having high-grade histology, it may be safely considered for FSS because of its great results.	[37]
Microsatellite Instability (MSI-H)	MMR-deficient (MMRd)	Intermediate	Frequently linked to Lynch syndrome, obesity, and younger age; variable grade	Given the moderate risk of recurrence, FSS may be provided with caution and requires close monitoring.	[38]
Copy Number Low (CN-low)	p53 wild-type (NSMP)	Intermediate	Typical endometrioid tumors, low-grade, hormone receptor positive	This group includes the majority of traditional FSS candidates; results are good but not remarkable.	[37]
Copy Number High (CN-high)	p53 abnormal (p53abn)	Poor	Serous/clear cell histology, older patients, aggressive course	Because of its poor survival rate and high recurrence rate, FSS is often contraindicated.	[36]

**Table 3:** Comparison of prognostic value of molecular subtypes across major studies (cohorts, percentages, CR, and recurrence rates) [65].

Molecular Subtype	Typical Cohort %	Complete Remission	Recurrence Rate	Prognosis
POLE-mutated	3–10%	>95%	<1–2%	excellent
MMRd	15–25%	85–90%	10–15%	intermediate
NSMP	35–55%	85–90%	10–15% (heterogenous)	variable
P53-abn	10–25%	60–70%	30–40%	poor

**Table 4:** Summary of clinical trials/studies evaluating molecular subtypes and response to conservative treatment.

Molecular Subtype	Complete Response to Progestins (6 mo)	Recurrence After Conservative Treatment	Clinical Implication	Level of Evidence	Limitations	References
POLE-mut	0%	50%	De-escalation feasible. FSS possible.	Retrospective cohort	Small sample size, multiple biases	[11,64,71,72]
MMRd	0%	100%	Lower response to progestin therapy. High recurrence after conservative treatment	Retrospective cohort	Small sample size, multiple biases	[64,71,72,73,74]
NSMP	24%	78%	Better response to conservative therapy than MMRd.	Retrospective cohort	Small sample size, multiple biases	[64,71,73]
P53abn	50%	0%	Conservative treatment contraindicated. Intensive therapy recommended.	Retrospective cohort	Small sample size, multiple biases	[11,64,71,73]

**Table 5:** A comparative synthesis table summarizing major reviews and guidelines [European Society of Gynecological Oncology (ESGO), European Society for Radiotherapy and Oncology (ESTRO)/European Society of Pathology (ESP), National Comprehensive Cancer Network (NCCN), ESMO] and how they address molecular profiling in fertility-sparing management.

Source/Guideline	Molecular Profiling Recommendation	Clinical Implication for FST	Patient Selection Criteria	Research Gaps/Notes	Reference
ESGO/ ESTRO/ ESPO	Recommends molecular classification for risk stratification; highlights POLE-mut, MMRd, NSMP, and p53abn subtypes	Suggests de-escalation for POLE-mut; close surveillance for MMRd; p53abn not ideal for FST	FST recommended for low-risk (stage IA, grade 1 endometrioid EC, ± focal LVI); FST may be considered for POLE-mut/NSMP; FST should be carefully evaluated for MMRd/p53abn for increased risk.	Data on FST outcomes by molecular subtype are limited; further trials needed	[85,86]
NCCN	Acknowledges molecular profiling as emerging; not yet standard for FST selection	No formal integration of molecular subtypes into FST recommendations	FST for grade 1, stage IA endometrioid EC; molecular data may inform but is not required	Calls for more evidence before routine use in FST	[86]
ESMO	Endorses molecular classification for prognosis; recognizes potential for FST selection	Encourages use of molecular data for counseling, especially for MMRd and p53abn	FST for early-stage, low-grade EC; molecular data may refine risk assessment	Emphasizes need for prospective studies	[86]
Major reviews (2021–2024)	Strongly advocate for molecular profiling (ProMisE/TCGA) to guide FST	Propose tailored FST: POLE-mut/NSMP candidates, MMRd/p53abn caution or exclusion	POLE-mut: favorable; NSMP: good prognosis; MMRd: higher recurrence; p53abn: poor prognosis	Highlight lack of robust data, especially for POLE-mut and p53abn in FST	[85,86,87]

**Table 6:** Summary of ongoing clinical trials assessing fertility-sparing strategies with molecular and fertility results in EC.

Trial (NCT No.)	Design	Population	Intervention	Primary Endpoints	Molecular Stratification	Fertility Outcomes Reported	References
feMMe trial (NCT01686126)	Phase II, randomized	Obese women with atypical hyperplasia or grade 1 EC	LNG-IUD ± metformin ± weight loss	Complete pathologic response at 6 months	Not integrated	Results of pregnancy are not primary; follow-up is exploratory	[13]
NCT01594879	Prospective, multicenter	Women < 40 with stage IA, grade 1 EC	LNG-IUD + oral MPA	Complete response rate	Not integrated	Fertility outcomes tracked (pregnancy rates)	[13,40]
NCT04008563	Prospective	Early-stage EC or atypical hyperplasia	Hysteroscopic resection + LNG-IUD	Recurrence-free survival	Not integrated	Pregnancy/live birth assessed as secondary	[40]
NCT03538704	Observational, prospective	Patients undergoing fertility-sparing treatment	Any progestin-based regimen	Treatment response	POLE, MMRd, p53-abn, NSMP	Fertility outcomes collected	[74]
NCT03932409	Phase II	Young women with grade 1 EC	Oral progestin + GnRH analog	Complete pathologic response	Not specified	Pregnancy/live birth not primary	[13]
Biomarker-driven prospective cohorts	observational	Early EC	Oral progestins or LNG-IUD	Molecular predictors of response, recurrence	ProMisE classifier (POLE, MMRd, p53, NSMP)	Fertility outcomes	[13,87]

## Data Availability

The original contributions presented in this study are included in the article. Further inquiries can be directed to the corresponding author.

## Abbreviations

The following abbreviations are used in this manuscript:

EC	Endometrial Cancer
AUB	Abnormal Uterine Bleeding
TH/BSO	Total hysterectomy with bilateral salpingo-oophorectomy
TCGA	The Cancer Genome Atlas
ProMisE	Proactive Molecular Risk Classifier for endometrial cancer
LVSI	Lymphovascular space invasion
D&C	dilation and curettage
MRI	Magnetic Resonance Imaging
TVUS	Transvaginal ultrasound
LNG-IUD	Norgestrel-Releasing Intrauterine Device
ECOG	Eastern Cooperative Oncology Group
CR	Complete response rate
RR	Recurrence rate
PR	Pregnancy rate
LBR	Live birth rate
ART	Assisted Reproductive Technology
MSI	microsatellite instability
CN-L	copy-number low
CN-H	copy-number high
POLE-mut	POLE-mutated
MMRd	mismatch repair deficient
p53-abn	p53 Abnormality
NSMP	non-specific molecular profile
IHC	immunohistochemistry
POLE	polymerase epsilon
EDM	exonuclease domains
FIGO	International Federation of Gynecology and Obstetrics
EEC	endometrioid carcinoma
MSI-high	Microsatellite instability-High
MMR	Mismatch repair
ESMO	European Society for Medical Oncology
PCR-based	molecular Polymerase Chain Reaction-based
ER	Estrogen Receptor
PR	Progesterone Receptor
PORTEC	Post Operative Radiation Therapy in Endometrial Carcinoma
NGS	Next-Generation Sequencing
LNG-IUS	Levonorgestrel-releasing Intrauterine System
ESGO	European Society of Gynaecological Oncology
ESTRO	European Society for Radiotherapy and Oncology
ESP	European Society of Pathology
NCCN	National Comprehensive Cancer Network
RCT	Randomized Controlled Trial
AI	Artificial Intelligence

## References

[1] World Cancer Research Fund Endometrial Cancer Statistics. https://www.wcrf.org/preventing-cancer/cancer-statistics/endometrial-cancer-statistics/.

[2] American Cancer Society Key Statistics for Endometrial Cancer. https://www.cancer.org/cancer/types/endometrial-cancer/about/key-statistics.html.

[3] Liu L., Habeshian T.S., Zhang J., Peeri N.C., Du M., De Vivo I., Setiawan V.W. (2023). Differential trends in rising endometrial cancer incidence by age, race, and ethnicity. JNCI Cancer Spectr..

[4] Guo F., Adekanmbi V., Hsu C.D., Hoang T.N., Soliman P.T., Baillargeon J.G., Berenson A.B. (2025). Trends in Endometrial Cancer Incidence Among Premenopausal and Postmenopausal Women in the United States Between 2001 and 2021. Cancers.

[5] Francoeur A.A., Liao C.-I., Chang J., Johnson C.R., Clair K., Tewari K.S., Kapp D.S., Chan J.K., Bristow R.E. (2024). Endometrial cancer and obesity trends in the United States in the 21st century. J. Clin. Oncol..

[6] Peeri N.C., Bertrand K.A., Na R., De Vivo I., Setiawan V.W., Seshan V.E., Alemany L., Chen Y., Clarke M.A., Clendenen T. (2025). Understanding risk factors for endometrial cancer in young women. JNCI J. Natl. Cancer Inst..

[7] Letourneau J.M., Ebbel E.E., Katz P.P., Katz A., Ai W.Z., Chien A.J., Melisko M.E., Cedars M.I., Rosen M.P. (2012). Pretreatment fertility counseling and fertility preservation improve quality of life in reproductive age women with cancer. Cancer.

[8] Clarke M.A., Long B.J., Del Mar Morillo A., Arbyn M., Bakkum-Gamez J.N., Wentzensen N. (2018). Association of Endometrial Cancer Risk with Postmenopausal Bleeding in Women: A Systematic Review and Meta-analysis. JAMA Intern. Med..

[9] Centini G., Colombi I., Ianes I., Perelli F., Ginetti A., Cannoni A., Habib N., Negre R.R., Martire F.G., Raimondo D. (2025). Fertility Sparing in Endometrial Cancer: Where Are We Now?. Cancers.

[10] Rodolakis A., Scambia G., Planchamp F., Acien M., Di Spiezio Sardo A., Farrugia M., Grynberg M., Pakiz M., Pavlakis K., Vermeulen N. (2023). ESGO/ESHRE/ESGE Guidelines for the fertility-sparing treatment of patients with endometrial carcinoma. Hum. Reprod. Open.

[11] Talhouk A., McConechy M.K., Leung S., Li-Chang H.H., Kwon J.S., Melnyk N., Yang W., Senz J., Boyd N., Karnezis A.N. (2015). A clinically applicable molecular-based classification for endometrial cancers. Br. J. Cancer.

[12] Jamieson A., McAlpine J.N. (2023). Molecular Profiling of Endometrial Cancer from TCGA to Clinical Practice. J. Natl. Compr. Cancer Netw..

[13] Obermair A., Baxter E., Brennan D.J., McAlpine J.N., Muellerer J.J., Amant F., van Gent M.D.J.M., Coleman R.L., Westin S.N., Yates M.S. (2020). Fertility-sparing treatment in early endometrial cancer: Current state and future strategies. Obstet. Gynecol. Sci..

[14] Garzon S., Uccella S., Zorzato P.C., Bosco M., Franchi M.P., Student V., Mariani A. (2021). Fertility-sparing management for endometrial cancer: Review of the literature. Minerva Med..

[15] Chandramohan A., Manchanda S., Renganathan R., Popat P.B., Shah D., Dhamija E., Sen A. (2024). Impact of the 2023 FIGO Staging System for Endometrial Cancer on the Use of Imaging Services: An Indian Perspective. Indian J. Radiol. Imaging.

[16] Dijkhuizen F.P., Mol B.W., Brölmann H.A., Heintz A.P. (2000). The accuracy of endometrial sampling in the diagnosis of patients with endometrial carcinoma and hyperplasia: A meta-analysis. Cancer.

[17] Ma W., Meng W., Yin J., Liang J., Wang X., Liu J., Shi F. (2025). Predictive value of models based on MRI radiomics and clinical indicators for lymphovascular space invasion in endometrial cancer. BMC Cancer.

[18] Kaketaka K., Tsuboyama T., Fukui H., Matsumoto S., Nakamoto A., Ota T., Honda T., Kiso K., Kido K., Tomiyama N. (2025). Assessment of endometrial cancer with microcystic, elongated, and fragmented pattern invasion using multiparametric MRI. Abdom. Radiol..

[19] Alcázar J.L., Orozco R., Martinez-Astorquiza Corral T., Juez L., Utrilla-Layna J., Mínguez J.A., Jurado M. (2015). Transvaginal ultrasound for preoperative assessment of myometrial invasion in patients with endometrial cancer: A systematic review and meta-analysis. Ultrasound Obstet. Gynecol..

[20] Yarandi F., Shirali E., Akhavan S., Nili F., Ramhormozian S. (2023). The impact of lymphovascular space invasion on survival in early stage low-grade endometrioid endometrial cancer. Eur. J. Med. Res..

[21] Concin N., Matias-Guiu X., Vergote I., Cibula D., Mirza M.R., Marnitz S., Ledermann J., Bosse T., Chargari C., Fagotti A. (2021). ESGO/ESTRO/ESP guidelines for the management of patients with endometrial carcinoma. Int. J. Gynecol. Cancer.

[22] Chen L., Xiao S., He S., Tian Q., Xue M. (2020). Factors That Impact Fertility after Hysteroscopic Adhesiolysis for Intrauterine Adhesions and Amenorrhea: A Retrospective Cohort Study. J. Minim. Invasive Gynecol..

[23] Zhang Y.-F., Fan Y., Mu Y., Li J.-K. (2023). Efficacy of Oral Medications or Intrauterine Device-Delivered Progestin in Patients with Endometrial Hyperplasia with or without Atypia: A Network Meta-Analysis. J. Clin. Med..

[24] Kesterson J.P., Fanning J. (2012). Fertility-sparing treatment of endometrial cancer: Options, outcomes and pitfalls. J. Gynecol. Oncol..

[25] Zhang X., Zhao X., Wang C., Lu S., Wang Y., He Y., Wang J., Shen D. (2023). Use of clinicopathological factors to predict prognosis of fertility-sparing treatment for endometrial endometrioid carcinoma and atypical hyperplasia. Oncol. Lett..

[26] Gao Y., Wang H., Jiang M., Cui Y., Yu X. (2025). Fertility-sparing treatment for patients with endometrial cancer: A bibliometric analysis from 2000 to 2024. Front. Oncol..

[27] Ogunbiyi M.O., Oxley S., Graham R., Olaitan A. (2024). The oncological and reproductive outcomes of fertility-preserving treatments for stage 1 grade 1 endometrial carcinoma: A systematic review and meta-analysis. J. Obstet. Gynaecol..

[28] Wei H., Pan N., Zhang W., Xiong G., Guo W., Dong Z., Ma C. (2023). Levonorgestrel-releasing intrauterine system-based therapies for early-stage endometrial cancer: A systematic review and meta-analysis. J. Gynecol. Oncol..

[29] Ye X., Li T. (2024). Effects of hysteroscopic surgery combined with progesterone therapy on fertility and prognosis in patients with early endometrial cancer and atypical endometrial hyperplasia or endometrial intraepithelial neoplasia: A meta-analysis. Arch. Gynecol. Obstet..

[30] Fan Y., Li X., Wang J., Wang Y., Tian L., Wang J. (2021). Analysis of pregnancy-associated factors after fertility-sparing therapy in young women with early stage endometrial cancer or atypical endometrial hyperplasia. Reprod. Biol. Endocrinol..

[31] Conroy R. (2023). Surgery/Progesterone Improves Responses, Pregnancy in Endometrial Cancers. https://www.cancernetwork.com/view/surgery-progesterone-improves-responses-pregnancy-in-endometrial-cancers.

[32] Xi Y., Liu G., Liu D., Jiang J., Gong R. (2023). Efficacy and pregnancy outcomes of hysteroscopic surgery combined with progestin as fertility-sparing therapy in patients with early stage endometrial cancer and atypical hyperplasia. Arch. Gynecol. Obstet..

[33] Chen J., Cao D. (2024). Fertility-sparing re-treatment for endometrial cancer and atypical endometrial hyperplasia patients with progestin-resistance: A retrospective analysis of 61 cases. World J. Surg. Oncol..

[34] Kandoth C., Schultz N., Cherniack A.D., Akbani R., Liu Y., Shen H., Robertson A.G., Pashtan I., Shen R., Cancer Genome Atlas Research Network (2013). Integrated genomic characterization of endometrial carcinoma. Nature.

[35] Yao X., Feng M., Wang W. (2024). The Clinical and Pathological Characteristics of POLE-Mutated Endometrial Cancer: A Comprehensive Review. Cancer Manag. Res..

[36] Agusti N., Kanbergs A., Nitecki R. (2024). Potential of molecular classification to guide fertility-sparing management among young patients with endometrial cancer. Gynecol. Oncol..

[37] Konecny G.E., Wang C., Hamidi H., Winterhoff B., Kalli K.R., Dering J., Ginther C., Chen H.-W., Dowdy S., Cliby W. (2014). Prognostic and Therapeutic Relevance of Molecular Subtypes in High-Grade Serous Ovarian Cancer. JNCI J. Natl. Cancer Inst..

[38] Mustea A., Ralser D.J., Egger E., Ziehm U., Vivas S., Brock S., Jackson D., Condic M., Meisel C., Otten L. (2023). Determination of the Cancer Genome Atlas (TCGA) Endometrial Cancer Molecular Subtypes Using the Variant Interpretation and Clinical Decision Support Software MH Guide. Cancers.

[39] Tanos V., Zervides Z., Tanos P. (2023). Prognostic Factors for Early-Stage Endometrial Cancer Management in Fertile Patients. Austin J. Obstet. Gynecol..

[40] Cavaliere A.F., Perelli F., Zaami S., D’Indinosante M., Turrini I., Giusti M., Gullo G., Vizzielli G., Mattei A., Scambia G. (2021). Fertility Sparing Treatments in Endometrial Cancer Patients: The Potential Role of the New Molecular Classification. Int. J. Mol. Sci..

[41] Temko D., Van Gool I.C., Rayner E., Glaire M., Makino S., Brown M., Chegwidden L., Palles C., Depreeuw J., Beggs A. (2018). Somatic POLE exonuclease domain mutations are early events in sporadic endometrial and colorectal carcinogenesis, determining driver mutational landscape, clonal neoantigen burden and immune response. J. Pathol..

[42] Tian W., Ji Z., Wang J., Meng J., Bi R., Ren Y., Shan B., Yang G., Wang H. (2022). Characterization of hotspot exonuclease domain mutations in the DNA polymerase ϵ gene in endometrial cancer. Front. Oncol..

[43] Vermij L., Jobsen J.J., León-Castillo A., Brinkhuis M., Roothaan S., Powell M.E., De Boer S.M., Khaw P., Mileshkin L.R., Fyles A. (2023). Prognostic refinement of NSMP high-risk endometrial cancers using oestrogen receptor immunohistochemistry. Br. J. Cancer.

[44] Kögl J., Pan T.L., Marth C., Zeimet A.G. (2024). The game-changing impact of POLE mutations in oncology—A review from a gynecologic oncology perspective. Front. Oncol..

[45] Meng B., Hoang L.N., McIntyre J.B., Duggan M.A., Nelson G.S., Lee C.-H., Köbel M. (2014). POLE exonuclease domain mutation predicts long progression-free survival in grade 3 endometrioid carcinoma of the endometrium. Gynecol. Oncol..

[46] Howitt B.E., Shukla S.A., Sholl L.M., Ritterhouse L.L., Watkins J.C., Rodig S., Stover E., Strickland K.C., D’Andrea A.D., Wu C.J. (2015). Association of Polymerase e–Mutated and Microsatellite-Instable Endometrial Cancers with Neoantigen Load, Number of Tumor-Infiltrating Lymphocytes, and Expression of PD-1 and PD-L1. JAMA Oncol..

[47] Kunkel T.A., Erie D.A. (2005). DNA Mismatch Repair. Annu. Rev. Biochem..

[48] Peltomäki P., Vasen H. (2004). Mutations Associated with HNPCC Predisposition—Update of ICG-HNPCC/INSiGHT Mutation Database. Dis. Markers.

[49] Adorisio R., Troncone G., Barberis M., Pepe F. (2024). Molecular Profiling of H-MSI/dMMR/for Endometrial Cancer Patients: “New Challenges in Diagnostic Routine Practice”. J. Mol. Pathol..

[50] Kanopiene D., Vidugiriene J., Valuckas K.P., Smailyte G., Uleckiene S., Bacher J. (2014). Endometrial cancer and microsatellite instability status. Open Med..

[51] Luchini C., Bibeau F., Ligtenberg M.J.L., Singh N., Nottegar A., Bosse T., Miller R., Riaz N., Douillard J.-Y., Andre F. (2019). ESMO recommendations on microsatellite instability testing for immunotherapy in cancer, and its relationship with PD-1/PD-L1 expression and tumour mutational burden: A systematic review-based approach. Ann. Oncol..

[52] Oaknin A., Gilbert L., Tinker A.V., Brown J., Mathews C., Press J., Sabatier R., O’Malley D.M., Samouelian V., Boni V. (2022). Safety and antitumor activity of dostarlimab in patients with advanced or recurrent DNA mismatch repair deficient/microsatellite instability-high (dMMR/MSI-H) or proficient/stable (MMRp/MSS) endometrial cancer: Interim results from GARNET-a phase I, single-arm study. J. Immunother. Cancer.

[53] Kasherman L., Ahrari S., Lheureux S. (2021). Dostarlimab in the Treatment of Recurrent or Primary Advanced Endometrial Cancer. Future Oncol..

[54] Marchetti M., Spagnol G., Vezzaro T., Bigardi S., De Tommasi O., Facchetti E., Tripepi M., Costeniero D., Munerol C., Maggino T. (2024). Low-Risk and High-Risk NSMPs: A Prognostic Subclassification of No Specific Molecular Profile Subtype of Endometrial Carcinomas. Cancers.

[55] Alafraidi M., Hoang L., Howitt B.E., Longacre T.A., McAlpine J.N., Jamieson A., Singh N., Gilks C.B., Pors J. (2024). The spectrum of oestrogen receptor expression in endometrial carcinomas of no specific molecular profile. Histopathology.

[56] Cole A.J., Dwight T., Gill A.J., Dickson K.-A., Zhu Y., Clarkson A., Gard G.B., Maidens J., Valmadre S., Clifton-Bligh R. (2016). Assessing mutant p53 in primary high-grade serous ovarian cancer using immunohistochemistry and massively parallel sequencing. Sci. Rep..

[57] Chang Y.-W., Kuo H.-L., Chen T.-C., Chen J., Lim L., Wang K.-L., Chen J.-R. (2024). Abnormal p53 expression is associated with poor outcomes in grade I or II, stage I, endometrioid carcinoma: A retrospective single-institute study. J. Gynecol. Oncol..

[58] Stelloo E., Nout R.A., Osse E.M., Jürgenliemk-Schulz I.J., Jobsen J.J., Lutgens L.C., van der Steen-Banasik E.M., Nijman H.W., Putter H., Bosse T. (2016). Improved Risk Assessment by Integrating Molecular and Clinicopathological Factors in Early-stage Endometrial Cancer-Combined Analysis of the PORTEC Cohorts. Clin. Cancer Res..

[59] Di Donato V., Iacobelli V., Schiavi M.C., Colagiovanni V., Pecorella I., Palaia I., Perniola G., Marchetti C., Musella A., Tomao F. (2018). Impact of Hormone Receptor Status and Ki-67 Expressionon Disease-Free Survival in Patients Affected by High-risk Endometrial Cancer. Int. J. Gynecol. Cancer.

[60] Baxter E., Brennan D.J., McAlpine J.N. (2021). Correction: Improving response to progestin treatment of low-grade endometrial cancer. Int. J. Gynecol. Cancer.

[61] Bostan I.-S., Mihaila M., Roman V., Radu N., Neagu M.T., Bostan M., Mehedintu C. (2024). Landscape of Endometrial Cancer: Molecular Mechanisms, Biomarkers, and Target Therapy. Cancers.

[62] Bosse T., ter Haar N.T., Seeber L.M., v Diest P.J., Hes F.J., Vasen H.F.A., Nout R.A., Creutzberg C.L., Morreau H., Smit V.T. (2013). Loss of ARID1A expression and its relationship with PI3K-Akt pathway alterations, TP53 and microsatellite instability in endometrial cancer. Mod. Pathol..

[63] Jiang F., Jiang S., Cao D., Mao M., Xiang Y. (2023). Immunologic Signatures across Molecular Subtypes and Potential Biomarkers for Sub-Stratification in Endometrial Cancer. Int. J. Mol. Sci..

[64] Wang J., Jiang G., Yan S., Tian Y., Jin Y., Fu H., Si L., Cai M., Liu X., Guo R. (2025). Molecular classification and fertility-sparing outcomes in endometrial cancer and atypical endometrial hyperplasia. Biomol. Biomed..

[65] Asami Y., Kobayashi Kato M., Hiranuma K., Matsuda M., Shimada Y., Ishikawa M., Koyama T., Komatsu M., Hamamoto R., Nagashima M. (2023). Utility of molecular subtypes and genetic alterations for evaluating clinical outcomes in 1029 patients with endometrial cancer. Br. J. Cancer.

[66] Thompson E.F., Huvila J., Jamieson A., Leung S., Lum A., Offman S., Lytwyn A., Sur M.L., Hoang L., Irving J. (2022). Variability in endometrial carcinoma pathology practice: Opportunities for improvement with molecular classification. Mod. Pathol..

[67] Vermij L., Smit V., Nout R., Bosse T. (2020). Incorporation of molecular characteristics into endometrial cancer management. Histopathology.

[68] Ferrari F.A., Uccella S., Franchi M., Scambia G., Fanfani F., Fagotti A., Pavone M., Raspagliesi F., Bogani G. (2025). Performance of molecular classification in predicting oncologic outcomes of fertility-sparing treatment for atypical endometrial hyperplasia and endometrial cancer. Int. J. Gynecol. Cancer.

[69] Hu Z., Wu Z., Liu W., Ning Y., Liu J., Ding W., Fan J., Cai S., Li Q., Li W. (2024). Proteogenomic insights into early-onset endometrioid endometrial carcinoma: Predictors for fertility-sparing therapy response. Nat. Genet..

[70] Symonds D.A. (1990). Prognostic value of pathologic features and DNA analysis in endometrial carcinoma. Gynecol. Oncol..

[71] Galant N., Krawczyk P., Monist M., Obara A., Gajek Ł., Grenda A., Nicoś M., Kalinka E., Milanowski J. (2024). Molecular Classification of Endometrial Cancer and Its Impact on Therapy Selection. Int. J. Mol. Sci..

[72] Janzen D.M., Rosales M.A., Paik D.Y., Lee D.S., Smith D.A., Witte O.N., Iruela-Arispe M.L., Memarzadeh S. (2013). Progesterone receptor signaling in the microenvironment of endometrial cancer influences its response to hormonal therapy. Cancer Res..

[73] Colombo N., Creutzberg C., Amant F., Bosse T., González-Martín A., Ledermann J., Marth C., Nout R., Querleu D., Mirza M.R. (2016). ESMO-ESGO-ESTRO Consensus Conference on Endometrial Cancer: Diagnosis, treatment and follow-up. Ann. Oncol..

[74] De Vitis L.A., Schivardi G., Delfrati S., Biffi B., Viscardi A., Rosanu M., Ribero L., Caruso G., Rappa A., Marinucci L. (2025). The prognostic impact of molecular classification in endometrial cancer that undergoes fertility-sparing treatment. Int. J. Gynecol. Cancer.

[75] Vitale S.G., Rossetti D., Tropea A., Biondi A., Laganà A.S. (2017). Fertility sparing surgery for stage IA type I and G2 endometrial cancer in reproductive-aged patients: Evidence-based approach and future perspectives. Updates Surg..

[76] Ronsini C., Mosca L., Iavarone I., Nicoletti R., Vinci D., Carotenuto R.M., Pasanisi F., Solazzo M.C., De Franciscis P., Torella M. (2022). Oncological outcomes in fertility-sparing treatment in stage IA-G2 endometrial cancer. Front. Oncol..

[77] Etrusco A., Laganà A.S., Chiantera V., Mikuš M., Arsalan H.M., d’Amati A., Vitagliano A., Cicinelli E., Favilli A., D’Amato A. (2024). Reproductive and Oncologic Outcomes in Young Women with Stage IA and Grade 2 Endometrial Carcinoma Undergoing Fertility-Sparing Treatment: A Systematic Review. Biomolecules.

[78] Schuurman T., Zilver S., Samuels S., Schats W., Amant F., van Trommel N., Lok C. (2021). Fertility-Sparing Surgery in Gynecologic Cancer: A Systematic Review. Cancers.

[79] Zhou L.-Z., Xiao H.-Q., Chen J. (2024). Mismatch repair gene MSH6 correlates with the prognosis, immune status and immune checkpoint inhibitors response of endometrial cancer. Front. Immunol..

[80] Tangen I.L., Werner H.M.J., Berg A., Halle M.K., Kusonmano K., Trovik J., Hoivik E.A., Mills G.B., Krakstad C., Salvesen H.B. (2014). Loss of progesterone receptor links to high proliferation and increases from primary to metastatic endometrial cancer lesions. Eur. J. Cancer.

[81] Bellone S., Roque D.M., Siegel E.R., Buza N., Hui P., Bonazzoli E., Guglielmi A., Zammataro L., Nagarkatti N., Zaidi S. (2022). A phase 2 evaluation of pembrolizumab for recurrent Lynch-like versus sporadic endometrial cancers with microsatellite instability. Cancer.

[82] Pandita P., Wang X., Jones D.E., Collins K., Hawkins S.M. (2019). Unique Molecular Features in High-Risk Histology Endometrial Cancers. Cancers.

[83] Mutlu L., Manavella D.D., Gullo G., McNamara B., Santin A.D., Patrizio P. (2022). Endometrial Cancer in Reproductive Age: Fertility-Sparing Approach and Reproductive Outcomes. Cancers.

[84] Tanos P., Dimitriou S., Gullo G., Tanos V. (2022). Biomolecular and Genetic Prognostic Factors That Can Facilitate Fertility-Sparing Treatment (FST) Decision Making in Early Stage Endometrial Cancer (ES-EC): A Systematic Review. Int. J. Mol. Sci..

[85] Bani M.A., Maulard A., Morice P., Chargari C., Genestie C. (2024). Integration of the Molecular Classification of Endometrial Carcinoma to Select Patients for Fertility Sparing Strategies. Anticancer Res..

[86] Navarria I., Usel M., Rapiti E., Neyroud-Caspar I., Pelte M.-F., Bouchardy C., Petignat P. (2009). Young patients with endometrial cancer: How many could be eligible for fertility-sparing treatment?. Gynecol. Oncol..

[87] Chung Y.S., Woo H.Y., Lee J.-Y., Park E., Nam E.J., Kim S., Kim S.W., Kim Y.T. (2021). Mismatch repair status influences response to fertility-sparing treatment of endometrial cancer. Am. J. Obstet. Gynecol..

[88] Fremond S., Andani S., Barkey Wolf J., Dijkstra J., Melsbach S., Jobsen J.J., Brinkhuis M., Roothaan S., Jurgenliemk-Schulz I., Lutgens L.C.H.W. (2023). Interpretable deep learning model to predict the molecular classification of endometrial cancer from haematoxylin and eosin-stained whole-slide images: A combined analysis of the PORTEC randomised trials and clinical cohorts. Lancet Digit. Health.

[89] Dagher C., Manning-Geist B., Ellenson L.H., Weigelt B., Rios-Doria E., Barry D., Abu-Rustum N.R., Leitao M.M., Mueller J.J. (2023). Molecular subtyping in endometrial cancer: A promising strategy to guide fertility preservation. Gynecol. Oncol..

[90] Contreras N.-A., Sabadell J., Verdaguer P., Julià C., Fernández-Montolí M.-E. (2022). Fertility-Sparing Approaches in Atypical Endometrial Hyperplasia and Endometrial Cancer Patients: Current Evidence and Future Directions. Int. J. Mol. Sci..

[91] Ansari N.M., Khalid U., Markov D., Bechev K., Aleksiev V., Markov G., Poryazova E. (2025). AI-Augmented Advances in the Diagnostic Approaches to Endometrial Cancer. Cancers.

[92] Xu Y., Zhao M., Zhang L., Wang T., Wang B., Xue Y., Xu Z., Shao W., Chen X., Wang C. (2023). Outcomes of fertility preservation treatments in patients with endometrial cancer with different molecular classifications based on an NGS panel. Front. Oncol..

[93] Bhardwaj V., Sharma A., Parambath S.V., Gul I., Zhang X., Lobie P.E., Qin P., Pandey V. (2022). Machine Learning for Endometrial Cancer Prediction and Prognostication. Front. Oncol..

[94] Stroeken Y., Hendriks F., Beltman J., Ter Kuile M. (2024). Quality of Life and Psychological Distress Related to Fertility and Pregnancy in AYAs Treated for Gynecological Cancer: A Systematic Review. Cancers.

[95] Emmert-Streib F., Manjang K., Dehmer M., Yli-Harja O., Auvinen A. (2021). Are There Limits in Explainability of Prognostic Biomarkers? Scrutinizing Biological Utility of Established Signatures. Cancers.

[96] Qian Y., Daza J., Itzel T., Betge J., Zhan T., Marmé F., Teufel A. (2021). Prognostic Cancer Gene Expression Signatures: Current Status and Challenges. Cells.

[97] Medford A.J., Moy B. (2024). Deficits of Molecular Prognosis/Diagnosis Studies in Underserved Populations. JCO Oncol. Pract..

[98] Gullo G., Etrusco A., Cucinella G., Perino A., Chiantera V., Laganà A.S., Tomaiuolo R., Vitagliano A., Giampaolino P., Noventa M. (2021). Fertility-Sparing Approach in Women Affected by Stage I and Low-Grade Endometrial Carcinoma: An Updated Overview. Int. J. Mol. Sci..

[99] Zhang T., Zhang X., Peng P., Yang J. (2024). Fertility-sparing treatment in MSI-H/MMRd endometrial carcinoma or atypical endometrial hyperplasia: A systematic review and meta-analysis. Eur. J. Obstet. Gynecol. Reprod. Biol..

[100] Peng S., Zheng Y., Liu J., Chen S., Yang K., Wang W., Ning G., Tang X., Li L., Ye Z. (2024). Molecular classification in fertility-sparing treatment of early-stage endometrial cancer: A potential tool for optimizing patient selection. Gynecol. Oncol..

[101] Koskas M., Uzan J., Luton D., Rouzier R., Daraï E. (2014). Prognostic factors of oncologic and reproductive outcomes in fertility-sparing management of endometrial atypical hyperplasia and adenocarcinoma: Systematic review and meta-analysis. Fertil. Steril..

[102] Gonçalves V., Ferreira P.L., Saleh M., Tamargo C., Quinn G.P. (2022). Perspectives of Young Women with Gynecologic Cancers on Fertility and Fertility Preservation: A Systematic Review. Oncologist.

[103] Giampaolino P., Cafasso V., Boccia D., Ascione M., Mercorio A., Viciglione F., Palumbo M., Serafino P., Buonfantino C., De Angelis M.C. (2022). Fertility-Sparing Approach in Patients with Endometrioid Endometrial Cancer Grade 2 Stage IA (FIGO): A Qualitative Systematic Review. BioMed Res. Int..

[104] Piergentili R., Gullo G., Basile G., Gulia C., Porrello A., Cucinella G., Marinelli E., Zaami S. (2023). Circulating miRNAs as a Tool for Early Diagnosis of Endometrial Cancer—Implications for the Fertility-Sparing Process: Clinical, Biological, and Legal Aspects. Int. J. Mol. Sci..

[105] Kuai D., Wei J., Li M., Chen L., Zhang D., Li X., He Y., Liu S., Zhang H., Tian W. (2024). Weight-Loss and Metformin-Use Improve the Reversal Rate in Patients with Endometrial Hyperplasia. Int. J. Women’s Health.

